# Smartphone-Based Psychotherapeutic Interventions in Blended Care of Cancer Survivors: Nested Randomized Clinical Trial

**DOI:** 10.2196/38515

**Published:** 2023-08-28

**Authors:** Gunther Meinlschmidt, Astrid Grossert, Cornelia Meffert, Noa Roemmel, Viviane Hess, Christoph Rochlitz, Miklos Pless, Sabina Hunziker, Brigitta Wössmer, Ulfried Geuter, Rainer Schaefert

**Affiliations:** 1 Department of Psychosomatic Medicine University Hospital Basel Basel Switzerland; 2 Faculty of Medicine University of Basel Basel Switzerland; 3 Department of Digital and Blended Psychosomatics and Psychotherapy Psychosomatic Medicine University Hospital Basel Basel Switzerland; 4 Division of Clinical Psychology and Cognitive Behavioral Therapy International Psychoanalytic University Berlin Berlin Germany; 5 Department of Medical Oncology University Hospital Basel Basel Switzerland; 6 Division of Clinical Psychology and Psychotherapy Department of Psychology University of Basel Basel Switzerland; 7 Medical Center of Oncology and Hematology Department of Psycho-Oncology Cantonal Hospital Baselland Liestal Switzerland; 8 Department of Medical Oncology Winterthur Cantonal Hospital Winterthur Switzerland; 9 Outpatient Practice for Psychotherapy Olten Switzerland; 10 Institute for Sports and Motology University of Marburg Marburg Germany

**Keywords:** digital therapeutics, ecological momentary assessment (EMA), ecological momentary intervention (EMI), internet- and mobile-based intervention, microintervention, neoplasm, smartphone-based intervention, postcancer treatment, body psychotherapy, mobile phone

## Abstract

**Background:**

Cancer is related to not only physical but also mental suffering. Notably, body image disturbances are highly relevant to cancer-related changes often persisting beyond recovery from cancer. Scalable and low-barrier interventions that can be blended with face-to-face psychotherapy for cancer survivors are highly warranted.

**Objective:**

The aim of the study is to investigate whether smartphone-based bodily interventions are more effective to improve the mood of patients with cancer than smartphone-based fairy tale interventions (control intervention).

**Methods:**

We recruited patients with cancer in 2 Swiss hospitals and conducted daily, fully automated smartphone-based interventions 6 times a week for 5 consecutive weeks, blended with weekly face-to-face group body psychotherapy. We applied 2 types of smartphone-based interventions using a within-subject design, randomly assigning patients daily to either bodily interventions or fairy tales. Each intervention type was presented 3 times a week. For this secondary analysis, 3-level mixed models were estimated with mood assessed by the 3 Multidimensional Mood Questionnaire subscales for good-bad mood, wakefulness, and calmness as key indicators. In addition, the effects on experience of presence, vitality, and burden assessed with visual analog scales were investigated.

**Results:**

Based on the data from *s*=732 interventions performed by 36 participants, good-bad mood improved (β=.27; 95% CI 0.062-0.483), and participants became calmer (β=.98; 95% CI 0.740-1.211) following smartphone-based interventions. Wakefulness did not significantly change from pre- to postsmartphone–based intervention (β=.17; 95% CI –0.081 to 0.412). This was true for both intervention types. There was no interaction effect of intervention type with change in good-bad mood (β=–.01; 95% CI –0.439 to 0.417), calmness (β=.22; 95% CI –0.228 to 0.728), or wakefulness (β=.14; 95% CI –0.354 to 0.644). Experience of presence (β=.34; 95% CI 0.271-0.417) and vitality (β=.35; 95% CI 0.268-0.426) increased from pre- to postsmartphone–based intervention, while experience of burden decreased (β=–0.40; 95% CI –0.481 to 0.311). Again, these effects were present for both intervention types. There were no significant interaction effects of intervention type with pre- to postintervention changes in experience of presence (β=.14; 95% CI –0.104 to 0.384), experience of vitality (β=.06; 95% CI –0.152 to 0.265), and experience of burden (β=–.16; 95% CI –0.358 to 0.017).

**Conclusions:**

Our results suggest that both smartphone-based audio-guided bodily interventions and fairy tales have the potential to improve the mood of cancer survivors.

**Trial Registration:**

ClinicalTrials.gov NCT03707548; https://clinicaltrials.gov/study/NCT03707548

**International Registered Report Identifier (IRRID):**

RR2-10.1186/s40359-019-0357-1

## Introduction

Cancer is an often life-threatening disease, posing multiple challenges. Although cancer is increasingly curable and the number of survivors has grown, it still remains one of the most feared diseases [1]. Patients living with cancer suffer from symptoms of their illness as well as from side effects of cancer therapies [2]. Both have physical but also mental implications, preventing patients from returning to their normal lives. Notably, body image disturbances are among the physically, mentally, and interpersonally most relevant cancer-related changes often persisting beyond initial recovery from cancer [3]. Key aspects of body image disturbances include (1) the self-perception of change in appearance and displeasure with this change, (2) a decline concerning various aspects of physical functioning, and (3) the psychological distress caused by these changes [4], highlighting the interrelatedness of body image disturbances with mood and affect. Considering these issues, developing interventions that target mental burden in posttreatment cancer survivors with bodily disturbances is highly warranted. Hence, we developed and applied a group body psychotherapy (BPT) for patients with cancer who are in posttreatment [5], which was based on an experience-oriented holistic approach [6,7].

Mobile mental health has become a topic of considerable interest for patients with cancer to promote self-management of their chronic disease [8]. Previous studies indicated that smartphone-based interventions have the potential to reduce symptoms of mental disorders, such as anxiety and depression [9,10]. Notably, smartphone-based interventions may be used as a specific type of ecological momentary intervention (EMI) and allow supporting patients in their daily lives, thereby reducing the personal and economic costs of mental health problems [11]. In addition, in the field of cancer treatment, there is an increasing focus on the development of technological at-home interventions that aim to improve health outcomes [12]. Furthermore, there is evidence that web-based interventions can be successfully blended with face-to-face psychotherapy [13] and that the use of mobile technology can increase the effectiveness of psychotherapeutic interventions [14]. Yet, the effects of smartphone-based interventions embedded in a psychotherapeutic context as blended psychotherapy for cancer survivors remain to be elucidated. Hence, we set out to complement group BPT by daily smartphone-based digital interventions, with the aim to investigate whether these had short-term effects on patients’ moods. We provided digital interventions based on daily randomization: either providing an intervention specifically addressing bodily perceptions consisting of bodily interventions or providing an unspecific intervention consisting of fairy tales as a comparator.

The goal of this randomized clinical trial component nested in a convergent parallel design was to explore changes in mood after smartphone-based bodily intervention compared to fairy tale intervention (comparator). It was hypothesized that the mood of cancer survivors improves from pre- to postsmartphone–based bodily interventions. Furthermore, we expected that mood improvement was greater following bodily interventions as compared to fairy tales (comparator). Due to a small study sample, we have performed exploratory analyses of our hypotheses.

## Methods

### Study Design and Setting

Presented data originated from a nested randomized controlled trial, embedded in a nonrandomized study registered in ClinicalTrials.gov (NCT03707548). The aim of this larger nonrandomized study was to evaluate the treatment effects of a BPT group intervention.

We recruited patients between September 3, 2018, and May 12, 2019, in 2 Swiss hospitals (University Hospital Basel and Cantonal Hospital Winterthur). All participants signed an informed consent before study participation. We kindly refer to a previous publication [15] for more information regarding the larger nonrandomized trial.

### Ethics Approval

The entire nonrandomized study, including the present nested randomized controlled trial component, is designed according to the Declaration of Helsinki, the Human Research Act, and the Human Research Ordinance. The Ethikkommission Zentral- und Nordwestschweiz (EKNZ; vote: EKNZ 2018-01115, dated August 28, 2018, and amendment dated March 14, 2019) has approved the study. In addition, we obtained ethical approval from the Kantonale Ethikkommission Zürich. Consistent with good clinical practice, we informed patients about participation in the larger nonrandomized study, the planned secondary analysis of data, and the implications of participation. All participants signed an informed consent form before study participation. Informed consent from the original, larger nonrandomized study allows the present analysis of secondary outcomes without additional consent. Participation was voluntary and could be withdrawn at any time during the entire study. Participants did not receive any compensation. Data were treated confidentially and were strictly analyzed in deidentified form.

### Inclusion Criteria and Recruitment

Inclusion criteria for the entire nonrandomized study were (1) age≥18 years, (2) sufficient knowledge of spoken German, (3) having received curatively intended treatment for any malignant neoplasm, (4) suffering from bodily disturbances, (5) primary treatment being completed at least 3 months prior to recruitment, (6) an Eastern Cooperative Oncology Group performance score of 0-1 [16], (7) an anticipated life expectancy of ≥12 months, and (8) the anticipated capacity to participate in the baseline assessment, the preintervention assessment, 6 group BPT sessions, the postintervention assessment, and the smartphone-based interventions and daily assessments. In addition, for participation in the smartphone-based component of the study, patients were required to own a smartphone and to be able to access their email accounts through it. Exclusion criteria for the entire nonrandomized study were (1) sign of progress or recurrence of malignancy at study inclusion, (2) having a severe current mental disorder, (3) risk of current suicidality, (4) participation in any other clinical trial with a psychosocial intervention, (5) receiving other current psychotherapeutic treatment for less than 6 months (with the exception of already existing therapies lasting ≥6 months), and (6) inability to understand and speak German. All eligibility criteria are described in detail in the study protocol [15]. Patients were recruited at the study centers; additionally, they were approached via public advertisements.

### Intervention

The smartphone-based digital intervention was embedded in a nonrandomized study with face-to-face psychotherapy, consisting of 6 group BPT sessions, 90 minutes each. As part of the nested randomized controlled trial component, participants received either an audio instruction of bodily interventions (3 times a week) or audio recordings of fairy tales as unspecific intervention and comparator (3 times a week) via smartphone between sessions, over a period of 5 consecutive weeks. There was no smartphone-based intervention on the day of the group BPT session. The smartphone-based bodily intervention offered audio clips consisting of BPT tools, experiences, and strategies that reflected the content of the face-to-face sessions. For more details on the contents of these bodily interventions, please refer to Multimedia Appendix 1 [7,15,17-19] or the entire study protocol [15]. The unspecific comparator interventions consisted of 15 selected Grimms’ fairy tales. Both types of interventions lasted about 10 minutes each. They were provided at random, with randomization taking place daily.

The Clinical Trial Unit of the University Hospital Basel independently generated the random sequences using R software (R Foundation for Statistical Computing), applying a block design to ensure that each patient received both interventions 3 times a week. This allowed individual daily randomization of each participant to either the bodily or the fairy tale intervention (within-subject randomization). Trial participants were blinded to randomization up until the moment at which the intervention was provided; body psychotherapists were also blinded to randomization.

To familiarize participants with the smartphone-based interventions, all patients received an invitational email with a link to an introductory audio file and the request to complete the questionnaire at the end of the first group BPT session. Data collected during this training were not included in the analyses.

Patients could freely choose the time of day they participated in the digital intervention. The time window started each day at 7 AM with the invitational email including the day-specific hyperlink giving access to the intervention. This hyperlink expired at midnight of the same day. We used on the web Questback software (Questback Ltd) [20] to conduct the smartphone-based interventions, including instructions, presentation of the audio clips, collection of the questionnaire data, and sending the invitational emails.

The detailed procedure of each smartphone-based intervention was as follows: (1) participants used their own smartphones to get connected via internet browser to the Questback server, using a day-specific personalized hyperlink provided in the daily invitational email. We instructed patients to log into their email once a day. (2) We asked patients to enter their individual self-generated personal code, which allowed for verifying subject identity. (3) Participants replied to a short questionnaire (“pre”) described in more detail below. (4) To start the session, patients were asked to click on the “play” button of the audio player. (5) Participants listened to the audio clip using either headphones or the smartphone speaker and eventually performed the bodily intervention. (6) Participants again replied to the short questionnaire (“post”). (7) The session finished by thanking the patients for their participation in that day’s session.

### Assessment

We assessed mood pre- and postsmartphone–based interventions via web-based questionnaires. We applied the German version of the “Multidimensional Mood Questionnaire” Short-Form A (MDMQ) [21,22]. The MDMQ Short-Form A comprises 12 adjectives, with three subscales: (1) good-bad mood, (2) awake-tired, and (3) calm-nervous. Each item is rated on a 5-point Likert scale ranging from 1=“not at all” to 5=“very.” For every subscale, we added up the values of the corresponding items, resulting in scale values potentially ranging between 4 and 20. High scores suggest positive affectivity, wakefulness, and calmness, respectively [21]. The MDMQ is a well-established tool for the self-assessment of current mood, especially suited for repeated measures with short intervals, which has previously been successfully applied within the context of smartphone-based microinterventions [23]. Additionally, we applied 3 single-item visual analog scales (VAS) to self-assess the experience of presence, vitality, and burden (eg, How present do you feel right now? VAS ranging from 0=”not at all” to 10=”extremely strong”).

We screened patients for eligibility at baseline assessment (T0), including standardized questionnaires and a semistructured interview. Included patients with cancer who are in posttreatment underwent a waiting period of approximately 6 weeks followed by a pre-face-to-face psychotherapy questionnaire assessment (T1). After the face-to-face group session, a postpsychotherapy assessment (T2) with standardized questionnaires and a second semistructured interview took place. The smartphone-based part of the study applied ecological momentary assessments taking place daily along the face-to-face psychotherapy sessions (ie, between T1 and T2).

### Statistical Analyses

**Figure 1:**
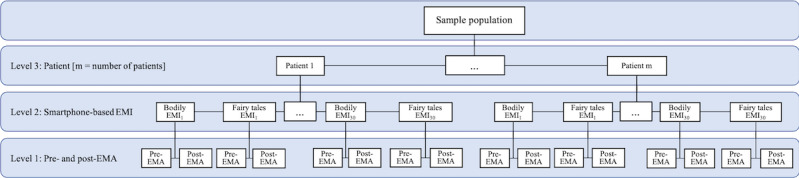
Mixed model diagram. Mixed model diagram for a 3-level hierarchical study with 2 types of smartphone-based interventions (bodily and fairy tales) nested in patients and pre- and postintervention assessments (based on the Logical Explanations & Visualizations of Estimates in Linear checklist by Monsalves et al [25]). EMA: ecological momentary assessment; EMI: ecological momentary intervention.

We conducted a secondary analysis of data collected in the larger nonrandomized study. The primary outcome analysis of the entire study is reported elsewhere [24]. According to Monsalves et al [25], calculating mixed models in a nested study design is indicated if the dependent variables are at a lower level than the independent variables. Hence, as we were interested in the effect of 2 different smartphone-based interventions (level 2) on mood changes in cancer survivors from pre- to postsmartphone–based intervention, we applied mixed models as indicated in Figure 1. To estimate changes in mood, experience of presence, experience of vitality, and experience of burden, mixed model analyses were conducted using restricted maximum likelihood (REML) estimation. Separate mixed models were calculated with the three MDMQ subscales: (1) good-bad mood, (2) awake-tired, and (3) calm-nervous as dependent variables. Similarly, separate mixed models were estimated with the single-item VAS experience of presence, experience of vitality, and experience of burden as dependent variables. Further, the main effect models included assessment time (pre- vs postsmartphone–based intervention) as an independent variable and interventions nested within individual participants as random intercepts. Interaction models included an interaction effect between assessment time (pre- vs postsmartphone–based intervention) and intervention type (fairy tales vs bodily interventions) as independent variables. Moreover, these cross-level interaction models included the lowest-level variable (pre- vs postsmartphone–based intervention) as random slopes, following suggestions by Heisig and Schaeffer [26]. Additionally, separate models were calculated for both smartphone-based intervention types (fairy tales and bodily interventions) with MDMQ subscales and the VAS for the experience of presence, vitality, and burden. We excluded subjects that did not participate in the smartphone-based component of the study and handled further missing data by applying mixed models with maximum likelihood estimation. For calculating and reporting mixed models, the Logical Explanations & Visualizations of Estimates in Linear mixed model checklist by Monsalves et al [25] was followed.

We compared the subsample of patients who participated in the smartphone-based intervention with the sample only participating in the larger nonrandomized study based on the variables age, gender, and distress at baseline (assessed by the National Comprehensive Cancer Network Distress Thermometer) using chi-square tests and *t* tests for independent samples. To investigate the association between age and frequency of participation in digital interventions, the Pearson correlation coefficient was calculated. We estimated gender-specific differences in participation in the digital interventions by using *t* tests for independent samples. The data for normal distribution by histograms and qq-plots were visually inspected. We summarized sample characteristics using descriptive statistics. We followed the CONSORT (Consolidated Standards of Reporting Trials) guidelines to report results (Multimedia Appendix 2).

We used R Studio (version 1.2.5033; R Foundation for Statistical Computing) [27] for all statistical analyses and visualization, importing the data into R Studio using the R package *haven* [28]. For data preparation and descriptive statistics, we used the R package *tidyverse* [29] in addition to basic R. The R package *lme4* [30] was used to conduct mixed model analyses, and the R package *effects* [31] was used to plot the models.

## Results

### Participant Characteristics

**Figure 2:**
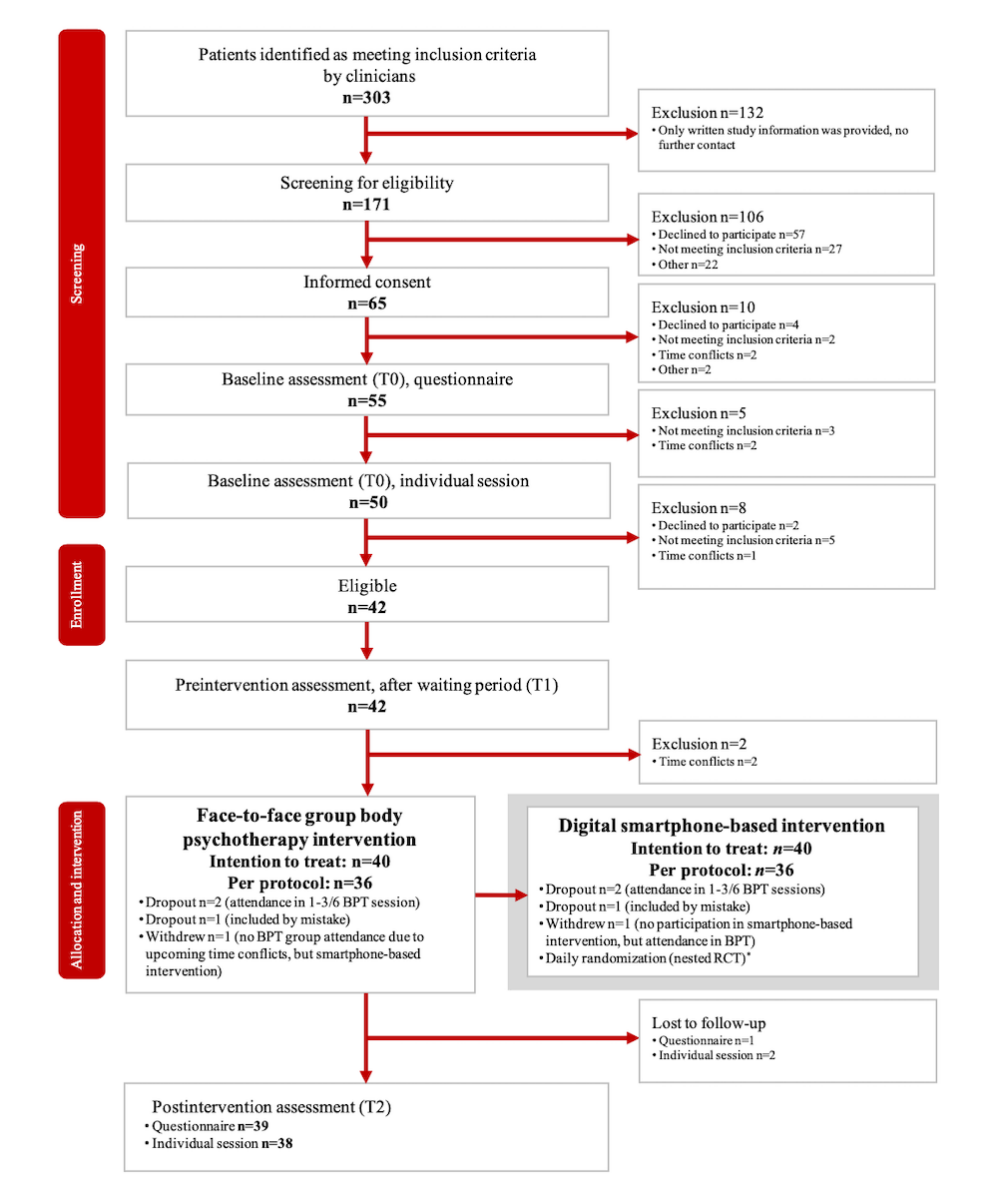
Study flow. *The digital smartphone-based bodily and fairy tale (comparator) interventions were provided over a period of 5 consecutive weeks on 6 days per week in parallel to the face-to-face group BPT phase. Thus, each patient underwent 15 bodily and 15 fairy tale interventions. BPT: body psychotherapy; RCT: randomized controlled trial.

We screened 171 patients, of whom 40 were scheduled to take part in the face-to-face group BPT (see the flowchart in Figure 2). In total, 39 of these patients met the inclusion criteria; 1 patient was included incorrectly. We formed 7 face-to-face psychotherapy groups, consisting of 5 to 7 patients each. Of the 40 patients scheduled to take part in the face-to-face group psychotherapy interventions of the larger nonrandomized study, 4 did not participate in the smartphone-based interventions and were thus excluded from this nested randomized controlled trial. One of the nonparticipants was the patient who had been included by mistake, 2 were dropout patients, and 1 patient took part in the group sessions but did not participate in the digital smartphone interventions. Another patient could not participate in the group sessions but agreed to take part in the smartphone-based intervention. Therefore, the results of the smartphone-based interventions are based on data from 36 participants. Participants and nonparticipants in the smartphone-based interventions did not differ significantly in terms of age (*P*=.70), gender (*P*=.43), and baseline distress (*P*=.44). Table 1 presents the sociodemographic and cancer-related characteristics of all patients participating in the nested randomized controlled trial, receiving the smartphone-based interventions.

**Table 1:** Sample characteristics.^a^

			Intention to treat (N=40)		Per protocol (N=36)
**Sex, n (%)**					
	Female	35 (87.5)		32 (88.9)	
	Male	5 (12.5)		4 (11.1)	
**Level of education, n (%)**					
	Elementary school	8 (20.5)		7 (22.9)	
	Secondary school	12 (30.8)		12 (31.4)	
	Technical college entrance qualification	8 (20.5)		5 (14.3)	
	High school graduation	8 (20.5)		8 (22.9)	
	Other certificates	3 (7.7)		3 (8.6)	
**Main diagnosis, n (%)**					
	MN^b^ of breast	23 (57.5)		22 (61.1)	
	Hodgkin lymphoma	4 (10.0)		4 (10.9)	
	Non-Hodgkin lymphoma	3 (7.5)		2 (5.6)	
	MN of lung	2 (5.0)		2 (5.6)	
	MN of ovary	1 (2.5)		1 (2.8)	
	MN of testis	1 (2.5)		1 (2.8)	
	MN of rectum	1 (2.5)		1 (2.8)	
	MN of small intestine	1 (2.5)		—^c^	
	MN of tongue	1 (2.5)		1 (2.8)	
	MN of kidney cell	1 (2.5)		—	
	MN of stomach	1 (2.5)		1 (2.8)	
	MN of peritoneum	1 (2.5)		1 (2.8)	
Age (range 22 to 77 years), mean (SD)			51.7 (13.8)		51.8 (14.4)

^a^Totals that do not add up to N=40 or N=36 are the result of missing values.

^b^MN: malignant neoplasm.

^c^Not available.

### Evaluation Outcomes

Results of our key secondary outcome variables assessed using the MDMQ scales indicate that postsmartphone–based intervention’s positive affectivity improved significantly (β=.27; 95% CI 0.062-0.483) and that patients became significantly calmer (β=.98; 95% CI 0.740-1.211; Table 2). However, participants did not experience significant changes in wakefulness pre- compared to postsmartphone–based intervention (β=.17; 95% CI –0.081 to 0.412). This was irrespective of the type of smartphone-based intervention. As depicted in Table 3, we did not find any interaction effect between the type of smartphone-based intervention and the change from pre- to postassessment for positive affectivity (β=–.01; 95% CI –0.439 to 0.417), calmness (β=.22; 95% CI –0.228 to 0.728), or wakefulness (β=.14; 95% CI –0.354 to 0.644). Similarly, the experience of presence (β=.34; 95% CI 0.271-0.417) and vitality (β=.35; 95% CI 0.268-0.426) increased significantly from pre- to postsmartphone–based intervention, while the experience of burden significantly decreased (β=–.40; 95% CI –0.481 to –0.311; Table 4). Again, these effects were independent of the type of smartphone-based intervention. As indicated in Table 5, there were no significant interaction effects between the type of smartphone-based intervention (bodily intervention vs fairy tale intervention) and the comparison of pre- and postassessment for the experience of presence (β=.14; 95% CI –0.104 to 0.384), the experience of vitality (β=.06; 95% CI –0.152 to 0.265), and the experience of burden (β=–0.16; 95% CI –0.358 to 0.017).

Furthermore, by calculating separate models for the 2 intervention types (Table 6), we found evidence that there was a significant increase in wakefulness in the bodily intervention (β=.25; 95% CI 0.050-0.442) but not in the comparator, fairy tales intervention (β=.09; 95% CI –0.109 to 0.290). In contrast, we found no significant pre- to postchanges of experience of presence, vitality, and burden when calculating separate models for the 2 smartphone-based intervention types (Table 7). The results of the effects related to the face-to-face BPT intervention will be reported elsewhere (personal communication by Grossert and colleagues, 2022).

**Table 2:** MDMQ^a^ random-intercept linear mixed models: main effects of pre- and postsmartphone–based intervention (N=36; models account for nested data [patient per intervention]).

Value of category		MDMQ good^b^ (*s*^c^=732)		MDMQ awake^d^ (*s*=732)		MDMQ calm^e^ (*s*=732)
**Pre- and postassessment level variables**						
	Preintervention	Reference	Reference		Reference	
	Postintervention, β (95% CI)	.27 (0.062 to 0.483)^f^	.17 (–0.081 to 0.412)		.98 (0.740 to 1.211)^f^	
ICC_patient_^g^		0.534		0.500		0.479
ICC_intervention_		0.032		0.043		0.041
AIC^h^		6436.23		6904.00		6756.88

^a^MDMQ: Multidimensional Mood Questionnaire.

^b^Intercept only model: intralevel correlation coefficient (ICC)_patient_=0.534; ICC_intervention_=0.032; Akaike information criterion (AIC)=6438.03.

^c^*s* is the number of successfully conducted interventions over all participants.

^d^Intercept only model: ICC_patient_=0.500; ICC_intervention_=0.043; AIC=6901.43.

^e^Intercept only model: ICC_patient_=0.470; ICC_intervention_=0.038; AIC=6817.11.

^f^Significant results.

^g^ICC: intralevel correlation coefficient.

^h^AIC: Akaike information criterion.

**Table 3:** MDMQ^a^ random-intercept and random-slope linear mixed models: interaction of intervention type and pre- and postsmartphone–based intervention (N=36; models account for nested data [patient per intervention]).

Value of category			MDMQ good^b^ (*s*^c^=732)		MDMQ awake^d^ (*s*=732)		MDMQ calm^e^ (*s*=732)
**Intervention-level variables**							
	Fairy tale (comparator)	Reference		Reference		Reference	
	Bodily intervention, β (95% CI)	.35 (–0.049 to 0.689)		.47 (–0.022 to 0.855)		.12 (–0.357 to 0.596)	
**Pre-and postlevel variables**							
	Preintervention	Reference		Reference		Reference	
	Postintervention, β (95% CI)	.28 (–0.021 to 0.577)		.09 (–0.257 to 0.441)		.86 (0.522 to 1.187)^f^	
**Cross-level interaction**							
	Intervention and pre-post, β (95% CI)	–.01 (–0.439 to 0.417)		.14 (–0.354 to 0.644)		.22 (–0.228 to 0.728)	
ICC_patient_^g^			0.568		0.537		0.550
ICC_intervention_			0.009		0.016		0.066
AIC^h^			6446.87		6923.23		6754.74

^a^MDMQ: Multidimensional Mood Questionnaire.

^b^Main effect model of intervention and pre-post: intralevel correlation coefficient (ICC)_patient_=0.569; ICC_intervention_=0.009; Akaike information criterion (AIC)=6450.40.

^c^*s* is the number of successfully conducted interventions over all participants.

^d^Main effect model of intervention and pre-post: ICC_patient_=0.537; ICC_intervention_=0.016; AIC=6920.78.

^e^Main effect model of intervention and pre-post: ICC_patient_=0.550; ICC_intervention_=0.066; AIC=6752.78.

^f^Significant results.

^g^ICC: intralevel correlation coefficient.

^h^AIC: Akaike information criterion.

**Table 4:** Visual analog scale random-intercept linear mixed models: main effects of pre- and postintervention (N=36; models account for nested data [patient per intervention]).

Value of category			Experience of presence^a^ (*s*^b^=732)		Experience of vitality^c^ (*s*=732)		Experience of burden^d^ (*s*=732)
**Pre- and postassessment level variables**							
	Preintervention	Reference		Reference		Reference	
	Postintervention, β (95% CI)	.34 (0.271 to 0.417)^e^		.35 (0.268 to 0.426)^e^		–.40 (–0.481 to –0.311)^e^	
ICC_patient_^f^			0.607		0.538		0.665
ICC_intervention_			0.039		0.060		0.030
AIC^g^			14,567.56		15,330.08		15,920.78

^a^Intercept only model: intralevel correlation coefficient (ICC)_patient_=0.603; ICC_intervention_=0.039; Akaike information criterion (AIC)=14,645.75.

^b^*s* is the number of successfully conducted interventions over all participants.

^c^Intercept only model: ICC_patient_=0.535; ICC_intervention_=0.060; AIC=15,396.17.

^d^Intercept only model: ICC_patient_=0.662; ICC_intervention_=0.029; AIC=15,997.08.

^e^Significant results.

^f^ICC: intralevel correlation coefficient.

^g^AIC: Akaike information criterion.

**Table 5:** Visual analog scale random-intercept and random-slope linear mixed models: interaction of intervention type and pre- and postintervention (N=36; models account for nested data [patient per intervention]).

Value of category		Experience of presence^a^ (*s*^b^=732)	Experience of vitality^c^ (*s*=732)	Experience of burden^d^ (*s*=732)
**Intervention-level variables**				
	Fairy tale (comparator)	Reference	Reference	Reference
	Bodily interventions, β (95% CI)	.05 (–0.161 to 0.254)	.08 (–0.168 to 0.319)	.02 (–0.244 to 0.276)
**Pre- and postlevel variables**				
	Preintervention	Reference	Reference	Reference
	Postintervention, β (95% CI)	.32 (0.120 to 0.515)^e^	.31 (0.151 to 0.478)^e^	–.34 (–0.522 to –0.159)^e^
**Cross-level interaction**				
	Intervention and pre-post, β (95% CI)	.14 (–0.104 to 0.384)	0.06 (–0.152 to 0.265)	–.16 (–0.358 to 0.017)
ICC_patient_^f^		0.570	0.551	0.655
ICC_intervention_		0.036	0.043	0.033
AIC^g^		14,479.56	15,316.24	15,902.87

^a^Main effect model of intervention and pre-post: intralevel correlation coefficient (ICC)_patient_=0.570; ICC_intervention_=0.036; Akaike information criterion (AIC)=14,476.51.

^b^*s* is the number of successfully conducted interventions over all participants.

^c^Main effect model of intervention and pre-post: ICC_patient_=0.551; ICC_intervention_=0.042; AIC=15,311.86.

^d^Main effect model of intervention and pre-post: ICC_patient_=0.654; ICC_intervention_=0.033; AIC=15,901.09.

^e^Significant results.

^f^ICC: intralevel correlation coefficient.

^g^AIC: Akaike information criterion.

**Table 6:** MDMQ^a^ random-intercept linear mixed models main effects of pre- and postintervention separately for bodily interventions and fairy tales interventions (comparator; N=36).

Value of category			MDMQ good^b^ (*s*^c^=732)	MDMQ awake^d^ (*s*=732)		MDMQ calm^e^ (*s*=732)
**Fairy tales (comparator)**						
	Preintervention	Reference		Reference	Reference	
	Postintervention, β (95% CI)	.27 (0.097 to 0.452)^f^		.09 (–0.109 to 0.290)	.85 (0.669 to 1.039)^f^	
	ICC_patient_^g^	0.479		0.531	0.004	
	ICC_intervention_	0.030		0.003	0.526	
	AIC^h^	10,063.42		10,593.79	10,260.67	
**Bodily interventions**						
	Preintervention	Reference		Reference	Reference	
	Postintervention, β (95% CI)	.27 (0.111 to 0.426)^f^		.25 (0.050 to 0.442)^f^	1.11 (0.914 to 1.300)^f^	
	ICC_patient_	0.367		0.121	0.013	
	ICC_intervention_	0.307		0.470	0.561	
	AIC	8812.84		9740.26	9622.30	

^a^MDMQ: Multidimensional Mood Questionnaire.

^b^Intercept only model of bodily interventions: intralevel correlation coefficient (ICC)_patient_=0.366; ICC_intervention_=0.307; Akaike information criterion (AIC)=8818.81; intercept only model of fairy tales: ICC_patient_=0.474; ICC_intervention_=0.034; AIC=10,067.62.

^c^*s* is the number of successfully conducted interventions over all participants.

^d^Intercept only model of bodily interventions: ICC patient=0.097; ICC_intervention_=0.494; AIC=9741.52; intercept only model of fairy tales: ICC_patient_=0.531; ICC_intervention_=0.003; AIC=10,589.84.

^e^Intercept only model of bodily interventions: ICC_patient_=0.038; ICC_intervention_=0.520; AIC=9742.46; intercept only model of fairy tales: ICC_patient_=0.002; ICC_intervention_=0.519; AIC=10,335.96.

^f^Significant results.

^g^ICC: intralevel correlation coefficient.

^h^AIC: Akaike information criterion.

**Table 7:** Visual analog scale random-intercept linear mixed models main effects of pre- and postintervention separately for bodily interventions and fairy tales interventions (comparator; N=36; models account for nested data [patient per intervention]).

Value of category		Experience of presence^a^ (*s*^b^=732)	Experience of vitality^c^ (*s*=732)	Experience of burden^d^ (*s*=732)
**Fairy tales (comparator)**				
	Preintervention	Reference	Reference	Reference
	Postintervention, β (95% CI)	.27 (0.177 to 0.368)^e^	.30 (0.194 to 0.415)^e^	–.32 (–0.440 to –0.200)^e^
	ICC_patient_^f^	0.406	0.220	0.335
	ICC_intervention_	0.285	0.402	0.332
	AIC^g^	7282.42	7946.26	8315.11
**Bodily interventions**				
	Preintervention	Reference	Reference	Reference
	Postintervention, β (95% CI)	.42 (0.310 to 0.532)^e^	.39 (0.278 to 0.507)^e^	–.48 (–0.598 to –0.357)^e^
	ICC_patient_	0.137	0.577	0.319
	ICC_intervention_	0.464	0.00003	0.405
	AIC	7331.91	7447.89	7691.17

^a^Intercept only model of bodily interventions: intralevel correlation coefficient (ICC)_patient_=0.139; ICC_intervention_=0.456; Akaike information criterion (AIC)=7380.44; intercept only model of fairy tales: ICC_patient_=0.387; ICC_intervention_=0.301; AIC=7307.48.

^b^*s* is the number of successfully conducted interventions over all participants.

^c^Intercept only model of bodily interventions: ICC_patient_=0.535; ICC_intervention_=0.037; AIC=7486.92; intercept only model of fairy tales: ICC_patient_=0.215; ICC_intervention_=0.405; AIC=7969.31.

^d^Intercept only model of bodily interventions: ICC_patient_=0.425; ICC_intervention_=0.294; AIC=7745.00; intercept only model of fairy tales: ICC_patient_=0.330; ICC_intervention_=0.334; AIC=8336.69.

^e^Significant results.

^f^ICC: intralevel correlation coefficient.

^g^AIC: Akaike information criterion.

Results of this secondary analysis were based on a total of *s*=732 interventions of 36 patients. These patients participated in 65.5% (354/540) of the smartphone-based bodily interventions and in 70% (378/540) of the smartphone-based control interventions (fairy tales). The frequency distribution of interventions per category over all patients is depicted in Multimedia Appendix 3. There were no statistically significant associations of the frequency of participation in the smartphone interventions with the age of the participants (*r*_34_=0.08; *P*=.64) and with gender (*r*_34_=–0.11; *P*=.23). Pre- and postsmartphone–based intervention, mean and SD of the MDMQ subscales and the 3 VAS items are depicted in Multimedia Appendix 4.

## Discussion

### Principal Results

The aim of this exploratory secondary analysis was to evaluate the potential of smartphone-based bodily interventions focusing on related mood changes from pre- to post-EMI in cancer survivors with body image disturbances. We compared smartphone-based bodily interventions with smartphone-based fairy tale interventions (comparator) using a within-subject design. Over the course of 5 consecutive weeks, participants were randomly assigned daily to either the bodily or fairy tale intervention (comparator). We blended face-to-face psychotherapy with this smartphone-based intervention. It was hypothesized that the mood of cancer survivors improves from pre- to postsmartphone–based bodily interventions. Furthermore, we expected that mood improvement was greater following bodily interventions as compared to fairy tales (comparator). Results indicate that the mood of patients with cancer who are in posttreatment improved following smartphone-based interventions, irrespective of the intervention type. Accordingly, results support the first part of our hypotheses but not the second. Hence, listening to fairy tales might have equally soothing and calming effects on people’s moods as bodily interventions [32]. Notably, the mere action of pausing daily life and listening to an audio clip might have positive effects on the general population and on cancer survivors’ moods. This phenomenon may in part also explain our findings that suggest the “active ingredients” of bodily interventions in the form of smartphone-based EMIs cannot fully explain mood improvements in cancer survivors. Further, in the context of the design of blended therapies, our study does not support the notion that the digital intervention component requires to be conceptually in line with the face-to-face intervention component [13].

In addition, we found no indication of an association between the patients’ age and the frequency of applying the smartphone-based intervention. Hence, there was no indication that younger patients were more skilled or motivated to use smartphone-based interventions as compared to older patients. Notably, the identified participation rates of between 65.5% and 70.0% can be seen as largely satisfactory, yet still indicate relevant potential for improvement, for example, by extending the time window in which patients were granted access to the daily digital interventions or by applying daily smartphone push notifications to remind patients to take part in the digital interventions. Importantly, mood differences from pre- to postsmartphone–based interventions were statistically significant but rather small in magnitude, indicating that a sequence of digital interventions with accumulating treatment effects [23] may be required to obtain clinically significant changes.

Overall, the findings of this study indicate that blending face-to-face BPT for cancer survivors with smartphone-based interventions is not only feasible—in line with previous reports on group therapy for depression [33], but is also likely to at least temporarily improve patients’ mood.

### Strengths and Limitations

Our study has several strengths. First, we used common technology (ie, no installation of special apps required) to provide daily and easily available body psychotherapeutic interventions, facilitating the uptake and translation of the interventions into routine clinical practice. Second, patients were free with regard to the timing of the smartphone-based interventions during everyday life, allowing a rather flexible integration of the smartphone-based intervention into daily routines. Third, smartphone-based interventions were designed to be very intuitive and straightforward to use, not requiring high internet or smartphone literacy of patients, further facilitating the uptake of the technology. Notably, the application of this kind of smartphone-based intervention could be particularly interesting for older patient populations and people with little smartphone or internet-related knowledge. Limitations of this study include a rather small number of included patients, which was only partially compensated by the up to 30 smartphone-based intervention sessions per patient. Our study was also limited by the fact that women with breast cancer were overrepresented. Although our group BPT was open to all patients with cancer who are in posttreatment with any malignant neoplasm, only 4 men participated. This should be taken into consideration when generalizing our findings. Furthermore, we could not blind participants with regard to the intervention. Hence, it is possible that patients were aware of what was the intervention of interest (bodily intervention) and what was the comparator (fairy tales). This may have resulted in biased mood assessments pre- and postsmartphone–based interventions. Nevertheless, we did not inform patients that the overall goal of the smartphone-based interventions was to compare the effects of bodily interventions with that of fairy tales on mood. Importantly, we measured changes in mood but not in bodily disturbances in relation to the smartphone-based interventions. Changes in bodily well-being were merely assessed at baseline (T0) and pre- (T1) and post- (T2) group face-to-face BPT intervention [15]. Thus, it remains unclear whether there were differences in effectiveness between the 2 smartphone-based interventions in terms of changing bodily disturbances or body mindfulness. Notably, fairy tales as an active comparator may have been a too powerful intervention strategy to detect significant differences. Furthermore, it was not possible to verify whether patients actually performed the smartphone-based bodily interventions or whether they just listened to the audio instructions. Thus, we could not distinguish between potential effects on mood, which resulted from merely listening to audio-guided bodily interventions and potential effects from performing the exercises. Finally, it is yet to be determined for how long the observed mood improvements following smartphone-based intervention persist in cancer survivors.

### Conclusions and Implications

The number of patients surviving cancer continues to rise. For example, there were 16.9 million cancer survivors in the United States on January 1, 2019 [34]. Many of them must cope with the physical effects of cancer and its treatment, potentially leading to functional, cognitive, and psychological impairments. Beyond that, in recent years, psychosocial interventions have gained increasing importance [35]. To further improve health-related quality of life in patients with cancer, innovative and scalable approaches are highly warranted.

The results of this study suggest that smartphone-based bodily interventions, which can be combined with face-to-face psychotherapy in terms of blended therapy may represent such an innovative intervention. This study underlines the feasibility and acceptance of smartphone-based interventions in postcancer survivors with bodily disturbances. These represent a new, promising treatment model that can be offered as a low-threshold supplement to face-to-face psychotherapy.

## Appendix

Multimedia Appendix 1 Content of interventions: group body psychotherapy with patients with cancer and smartphone-based bodily interventions (published in Grossert et al [15]).

Multimedia Appendix 2 CONSORT-eHEALTH checklist (V 1.6.1).

Multimedia Appendix 3 Distribution of intervention frequency (number of interventions).

Multimedia Appendix 4 Descriptive values of pre- and postsmartphone–based intervention assessments.

## Abbreviations

BPT: body psychotherapy
CONSORT: Consolidated Standards of Reporting Trials
EKNZ: Ethikkommission Nord und Zentralschweiz
EMI: ecological momentary intervention
MDMQ: Multidimensional Mood Questionnaire
REML: restricted maximum likelihood
VAS: visual analog scale

## Data Availability

Due to the nature of this research, participants of this study did not agree for their data to be shared publicly; hence, supporting data are not available.
