# Supplementary material for: Smartphone-Based Psychotherapeutic Interventions in Blended Care of Cancer Survivors: Nested Randomized Clinical Trial
Source: JMIR Cancer. 2023 Aug 28;9:e38515. doi: 10.2196/38515 (PMC10495843; doi:10.2196/38515)
Supplement: Multimedia Appendix 1 [file cancer_v9i1e38515_app1.docx]

**Multimedia Appendix 1.** Content of interventions: group body psychotherapy with patients with cancer and smartphone-based bodily interventions (published in Grossert et al [15])

|  | **Content group body psychotherapy** | | | | **Content audiofiles** |
| --- | --- | --- | --- | --- | --- |
| **Session** | **Topic** | **Opening**  **including short feedback on the previous session** | **Introduction & Exercise including sharing**   1. **Introduction and Psychoeducation** 2. **Strategies/Exercise** 3. **Sharing of own experiences** | **Closure**  **including perspective for the upcoming week** | **Smartphone based bodily interventions**  **supporting transfer from group BPT sessions into daily live** |
| **Duration** | **In Total 90 min** | **15-20 min** | **50-65 mins** | **10-15 min** | randomly on 3 days a week  10-15 min |
| 1. | **Group cohesion and body perception and awareness** | - Self-introduction - Expectations and fears | 1. - Reflection about bodily perception, body image, body disturbances and body work experience - Introduction of BPT terms 2. - Breath perception  - Body awareness with BodyScan technique, supine position - Self - contact (hands on/off) 3. Reflection about own experience during the exercises | What can I take with me after this first contact in the group? | Body awareness with body scan technique |
| 2. | **Bodily resources and**  **grounding/ anchoring** | - Short body scan CEB - Breath perception - Feedback | 1. - Body as a resource   - Balance between distress and resources, bodily stress reaction, adapted [17]   1. - Body awareness with BodyScan technique, standing position   - Foot work and anchoring with the focus on connection to stability, e.g. p97 [7] - Movement perception including mirroring   1. Reflection about own experience during the exercises | How to transfer exercise skills into daily life? | Anchoring/grounding with footwork using a small rubber ball |
| 3. | **Boundary awareness** | - Short body scan CEB - Anchoring exercise - Feedback | 1. Space and boundaries including importance of having the choice between own and shared space 2. Boundary awareness:   - Lika Breathing technique, p37 [18]  - Boundary awareness: exploring own space and own boundaries, p84 [7]   1. Reflection about own experience during the exercises | Transfer of boundary awareness into daily life experience. | Boundary awareness through Lika breathing technique |
| 4. | **Impulses and social/group interaction** | - Short body scan CEB - Lika breathing technique, p37 (4) - Feedback | 1. Social interactions and (body) impulses to get into/out of social interactions 2. – Body self-release techniques, adapted p209 [7]   - Awareness of bodily perception and the nature of impulses  - nonverbal contact Interaction with different body parts   1. Reflection about own experience during the exercises | How can I find a witness position being aware of perception and impulses?  What do I need? | Relaxation through body self-release techniques |
| 5. | **Embodied emotions** | - Short body scan CEB - Anchoring exercise - Feedback | 1. Integration model of human experience, p26 [7] 2. - Mapping of feelings under the cancer disease and treatment   - Focus on embodiment: body sculptures of emotions, adapted [19]   1. Reflection about own experience during the exercises | How to become aware of feelings and how to explore and share them in daily life? | Self-awareness through ‘four body zone’ exercise |
| 6. | **Summary** | - Short body scan CEB - Free choice of exercise repetition - Feedback | 1. Summary and open questions 2. Free choice of exercise repetition 3. Closing: Ritual “*being connected while continue on individual path*” | What would I take with me?  What would I leave in this group/room?  Evaluation |  |

*BPT: Body Psychotherapy, CEB: cognition, emotions, body perception*
