# Supplementary figures and images for: Smartphone-Based Psychotherapeutic Interventions in Blended Care of Cancer Survivors: Nested Randomized Clinical Trial
Source: JMIR Cancer. 2023 Aug 28;9:e38515. doi: 10.2196/38515 (PMC10495843; doi:10.2196/38515)

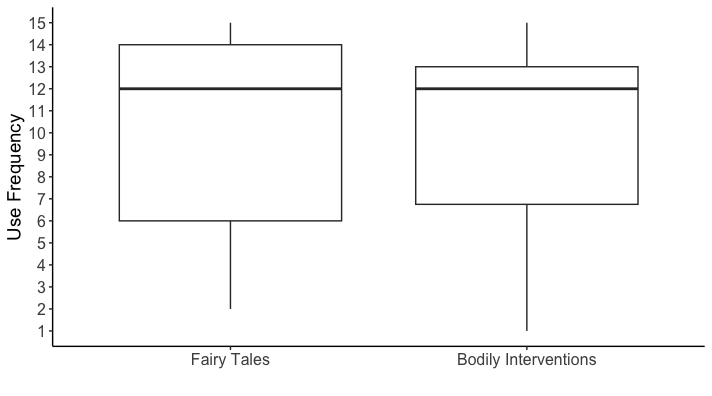

Supplement: Multimedia Appendix 3 [file cancer_v9i1e38515_app3.png]
