# Supplementary material for: Smartphone-Based Psychotherapeutic Interventions in Blended Care of Cancer Survivors: Nested Randomized Clinical Trial
Source: JMIR Cancer. 2023 Aug 28;9:e38515. doi: 10.2196/38515 (PMC10495843; doi:10.2196/38515)
Supplement: Multimedia Appendix 4 [file cancer_v9i1e38515_app4.docx]

**Multimedia Appendix 4.** Descriptive values of pre- and postsmartphone–based intervention assessments.

|  | Pre-EMI | | Post-EMI | |
| --- | --- | --- | --- | --- |
|  | *Mean* | *SD* | *Mean* | *SD* |
| **Fairy tales** | | | | |
| MDMQ Good | 15.13 | 3.18 | 15.40 | 2.99 |
| MDMQ Awake | 12.05 | 3.60 | 12.13 | 3.48 |
| MDMQ Calm | 13.67 | 3.40 | 14.52 | 3.16 |
| Experience of presence | 6.29 | 2.00 | 6.56 | 2.12 |
| Experience of vitality | 5.81 | 2.15 | 6.12 | 2.10 |
| Experience of burden | 4.05 | 2.41 | 3.73 | 2.33 |
|  |  |  |  |  |
| **Bodily interventions** | | | | |
| MDMQ Good | 15.63 | 3.06 | 15.90 | 3.01 |
| MDMQ Awake | 12.62 | 3.66 | 12.86 | 3.59 |
| MDMQ Calm | 13.93 | 3.51 | 15.04 | 3.23 |
| Experience of presence | 6.46 | 2.10 | 6.88 | 2.03 |
| Experience of vitality | 6.04 | 2.08 | 6.43 | 2.00 |
| Experience of burden | 3.91 | 2.45 | 3.43 | 2.32 |

*Notes. Values for MDMQ subscales range from four to 20. Values for VAS of experience of presence, vitality, and burden range from zero to 10.*

*Abbreviations. EMI, Ecological Momentary Intervention (= smartphone-based digital interventions, namely fairy tales and bodily interventions); MDMQ, Multidimensional Mood Questionnaire; SD, standard deviation.*
